# Post-hysterectomy vaginal cuff cancer secondary to HPV infection and CIN: A case report

**DOI:** 10.12669/pjms.294.3073

**Published:** 2013

**Authors:** Xiaoxia Liu, Ying Yue, Shan Zong

**Affiliations:** 1 Xiaoxia Liu, PhD, Obstetrics and Gynecology Center, First Hospital of Jilin University Norman Bethune, Changchun, 130021, China.; 2 Ying Yue, PhD, Obstetrics and Gynecology Center, First Hospital of Jilin University Norman Bethune, Changchun, 130021, China.; 3 Shan Zong, MD, Obstetrics and Gynecology Center, First Hospital of Jilin University Norman Bethune, Changchun, 130021, China.

**Keywords:** Cervical intraepithelial neoplasia, Human papillomavirus, Vaginal intraepithelial neoplasia, Vaginal cancer

## Abstract

We present a case report of secondary vaginal cancer after complete hysterectomy due to myoma that was complicated by low-grade cervical intraepithelial neoplasia (CIN I) and human papilloma virus (HPV) infection. After complete hysterectomy, the HPV DNA level in the vaginal tissue was obviously increased, and vaginal cancer was diagnosed 6 months later. We conclude that HPV infection can cause vaginal cancer after complete hysterectomy in cases complicated by CIN. Therefore, HPV should be regularly assessed during the postoperative follow-up period.

## INTRODUCTION

The incidence of vaginal intraepithelial neoplasia (VAIN) and vaginal cancer is very low, at 0.2-0.3 and 0.42 per 100,000, respectively, in the USA.^1^ Conclusive evidence has demonstrated that a large number of cervical cancers occur due to infection with HPV, of which strains HPV-16 and HPV-18 are particularly important. HPV viral subtypes can also cause other cancers, including vulval, vaginal, anal, penile and oropharyngeal carcinomas.^2^ Women over 60 years of age who have CIN lesions are at an elevated risk, and should be followed closely. Follow-up should continue even after a hysterectomy.^3^ Here, we present a case report of secondary vaginal cancer after complete hysterectomy due to myoma, which was complicated by low-grade cervical intraepithelial neoplasia (CIN I) and human papilloma virus (HPV) infection. A review of the relevant literature is presented to increase awareness of the importance of monitoring HPV infection.

## CASE PRESENTATION

**Fig.1:**
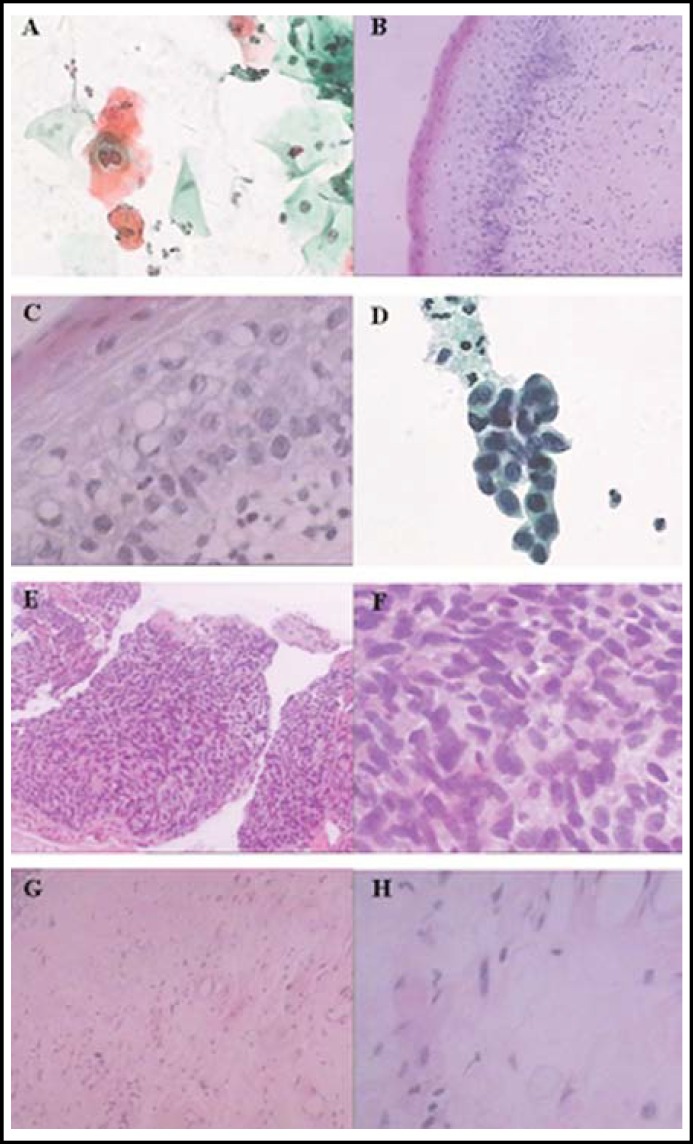
** (A)** Low-grade squamous intraepithelial lesions (LSILs) of the cervix. **(B)** CIN I (magnification ×100). **(C)** CIN I (magnification ×400). **(D)** High-grade vaginal intraepithelial neoplasia (VAIN III). **(E)** Vaginal cancer (×100). **(F)** Vaginal cancer (×400). **(G)** Vaginal biopsy after radiotherapy (×100). **(H)** Vaginal biopsy after radiotherapy (×400).

A 62-year-old para 2, gravida 2 married patient was admitted to our unit due to intermittent vaginal bleeding over the preceding year. Four months prior to admission, she had undergone a thin-prep cytological test (TCT) which revealed low-grade squamous intraepithelial lesions (LSILs) of the cervix (Fig.1A). Her hybrid capture 2-human papillomavirus (HC2-HPV-DNA) test values had increased significantly from 8.13 pg/ml to 673.69 pg/ml after a one-month interval. The pathological results from colposcopy and biopsy showed chronic cervicitis. The patient had no history of genital warts, radiotherapy, or immunosuppression.

The patient was found to have uterine fibroids, and these were treated surgically. After anesthesia was instituted, a vaginal conventional hysterectomy was performed. Histopathological examination of the resected uterus revealed uterine fibroids, endometrial atrophy, chronic cervical inflammation, squamous cell hyperplasia, localized low-grade CIN I, and interstitial gland tumor-like hyperplasia (Fig.1B, C). The short-term follow-up of the patient revealed no clinical symptoms, such as vaginal bleeding.

The patient was reviewed in clinic 6 months post-operatively. A vaginal cytology test by the TCT method showed dyskaryosis, with grade III high-grade squamous vaginal intraepithelial neoplasia (Fig.1D). The result of a repeat HC2-HPV-DNA investigation was 1040.6 pg/ml. Colposcopy revealed macroscopic white lesions on the left vaginal cuff that measured approximately 0.2 cm in diameter; biopsies of these lesions revealed poorly differentiated invasive squamous cell carcinoma (Fig.1E, F). In this case, hysterectomy was performed for CIN I and the final pathological findings showed a normal vaginal resection margin. Six months later, poorly differentiated vaginal squamous cell carcinoma was diagnosed to be at FIGO stage I. The patient opted for radiotherapy in order to avoid a second surgical procedure, and this took place a week after diagnosis. As the squamous carcinoma lesions were located in the upper third of the vagina, the dose (70 Gy, 35 doses) and location of pelvic radiation was almost identical to that used for cervical cancer. Two months after radiotherapy, follow-up colposcopy with biopsies showed no tumor tissue or recurrence (Fig.1G, H). The patient remains tumor-free and in good health.

## DISCUSSION

VAIN and vaginal cancers are rare diseases with unclear epidemiologies. Conclusive evidence has demonstrated that a large number of cervical cancers occur due to infection with HPV.^2^ Brinton et al found that low education, low income, previous abnormal Pap smears, genital warts and a history of hysterectomy were important risk factors for VAIN and vaginal cancer; other risk factors included a current or past history of CIN, immunosuppression, previous radiotherapy and exposure to diethylstilbestrol.^4^

A history of hysterectomy for CIN is a known risk factor for secondary VAIN; the incidence of VAIN ranges between 0.9%–6.8% after hysterectomy for CIN.^5^ The outcomes of conservative treatment with cervical cone excision and total hysterectomy are similar. Therefore, to reduce the increased risk of injury associated with more extensive surgery, a hysterectomy for CIN II or CIN III is not usually indicated.^5^ We report the case of an elderly women with persistent HPV infection and CIN I who presented with vaginal bleeding secondary to uterine fibroids. She accepted the surgical intervention of hysterectomy with the aim of preventing future cervical cancer. Due to the patient’s age, and the high HPV viral load, we investigated her postoperatively with testing of vaginal exfoliated cells for HC2-HPV DNA. Vaginal cancer was identified after six months; with timely treatment, and her condition was well-controlled. Therefore, we advocate that patients with CIN, including those who have undergone hysterectomy, should undergo cervical conization and routine HC2-HPV DNA testing.

Schockaert et al^5^ recommended that cervical and vaginal colposcopy should be performed prior to hysterectomy due to CIN. The histology of exfoliated vaginal wall cells should be followed-up postoperatively for at least 18 months with samples taken every six months. If the histology at 18 months is negative for carcinoma, then follow-up can be discontinued.^6^ After treatment, colposcopy should be re-examined each year for up to 4 years after surgery. Patients with CIN I should be followed with annual colposcopy inspections for 2 years while a 10 year follow-up is recommended for patients with CIN II-III. This policy of post-treatment cytological surveillance ensures early detection of treatment failure and repeat conservative treatment as necessary for those who have a preserved uterus with cervical conization. However, some scholars believe that vaginal cancer does not develop after the hysterectomy, but the primary cancer in the vagina was already present prior to surgery. Therefore, a full examination should be conducted prior to the hysterectomy to prevent a missed diagnosis. 

In conclusion, HPV infection is the cause of cervical cancer, vaginal cancer, vulvar cancer, VAIN and other diseases. HPV testing has a powerful negative predictive value; if patients are followed-up for 6 months, a negative HC2-HPV-DNA test indicates that the risk of CIN recurrence is very small.^2^ HC2-HPV-DNA testing is crucial for the successful overall outcome of conservative management of cervical CIN or in those who undergo total hysterectomy. Therefore, for resections due to CIN by cervical cone or hysterectomy patients, follow-up should include the HC2-HPV-DNA test and TCT, with HC2-HPV-DNA testing given higher priority. If the HC2-HPV-DNA test is positive, she should undergo cytology and, if abnormal cytology is present, colposcopy can then assist with the early detection of disease recurrence. If colposcopy is negative for HC2-HPV-DNA on the initial examination, repeated HC2-HPV-DNA testing and cytology inspection at 12 months will be adequate to discern disease recurrence. We believe that persistent HPV infection is the most salient risk factor for the recurrence of cervical CIN, from which VAIN and vaginal cancer can develop. The decision to commence antiviral therapy and the choice of therapy are therefore important questions that require accurate diagnostic tools and follow-up.

## Notes

***Declaration of interest:*** There was no conflict of interest.
